# Intra- and intermuscular variations of postmortem protein degradation for PMI estimation

**DOI:** 10.1007/s00414-020-02355-4

**Published:** 2020-07-07

**Authors:** Stefan Pittner, Walther Gotsmy, Angela Zissler, Bianca Ehrenfellner, Dominik Baumgartner, Anna Schrüfer, Peter Steinbacher, Fabio Monticelli

**Affiliations:** 1 Department of Forensic Medicine, University of Salzburg, Salzburg, Austria; 2 Department of Biosciences, University of Salzburg, Salzburg, Austria

**Keywords:** PMI, Muscle, Protein, Degradation, Skeletal, Cardiac, Smooth

## Abstract

In recent years, protein decomposition has become of increasing interest for the use in forensic estimation of the postmortem interval (PMI). Especially skeletal muscle tissue has proven to be a prime target tissue, among other reasons, due to its large abundance in the human body. In this regard, it is important to know whether there are any intra- and intermuscular differences in the behavior of protein degradation. Thus, samples from different locations within several skeletal muscles as well as from cardiac and smooth muscle tissue samples were collected from three autopsy cases with varying degree of decomposition. Samples were analyzed by SDS-PAGE and Western blotting and compared for protein degradation patterns. Intramuscular variations turned out to be minimal and without major influence for the use of the method. Observed intermuscular differences provide possibilities for future improvement of the precision and temporal application range. The results of this study show the strengths and current limitations of protein degradation-based PMI estimation and provide a deeper understanding of intraindividual postmortem protein degradation processes.

## Introduction

Time since death estimation is a crucial aspect in forensic routine and yet often remains unsuccessful considering the currently available methodical spectrum. In recent years, several approaches based on postmortem decomposition of biomolecules, particularly proteins, have been suggested for additional delimitation of the postmortem interval (PMI) [1–3]. Although there are numerous aspects to be investigated, possibly affecting the outcome of the analysis and thus the accuracy and reliability of the method, some approaches appear promising candidates for future casework.

Especially in skeletal muscle, protein degradation has been extensively investigated, depicting beneficial assets as a target tissue. Skeletal muscle tissue is well protected from environmental influences and at the same time easy to access and to handle [1]. Additionally, it represents the largest homogenous compartment of the human body, contributing to 30–40% of bodyweight [4]. This constitutes a major advantage in both research (e.g., multiple samples can be taken and analyzed, without interference by previous samplings) and practical application (e.g., even in severely injured bodies or body parts, there is a high chance to be able to collect unaffected tissue for analysis). Available data, however, is largely limited to thigh muscle [5–8], with few exceptions (e.g., *M. psoas* [9], *M. gastrocnemius* [10]). To fully benefit from the described advantages of muscle tissue, further research is required regarding decomposition similarities and deviations within individual muscles, between muscles, and between muscle types (skeletal muscle, cardiac and smooth muscle). If there is no difference in degradation patterns of muscle tissue, a highly conserved mechanism of decomposition is indicated supporting the opportunity to sample any (type of) muscle available for forensic PMI estimation. If, however, differences between muscles are detected, there could be a possibility for succession patterns: If a degradation event has occurred in muscle A but not (yet) in muscle B, more precise PMI estimations could be possible when several muscles are analyzed. Additionally, the temporal range of the method could eventually be extended.

Considering that physical circumstances (e.g., temperature dependence of the postmortem breakdown of proteins [1, 5] and differential cooling of body compartments [11]) as well as physiological aspects (e.g., in vivo variations [7]) can affect degradation, inter- and intramuscular deviations can also be expected in humans. Since different muscle groups have different proportions of muscle fiber types, decomposition patterns might deviate, as faster degradation of muscle proteins in type II fibers compared with type I fibers had been demonstrated in pigs [12]. Within an individual muscle, similar variations can be caused by a larger share of type I muscle fibers in deeper regions and in the close proximity to bones and tendons [13, 14]. The vicinity to a tendon might also alter data outcome. Increased amounts of collagen (connective tissue) close to myotendinous junctions [15] can entail lower content of target proteins in the sampled muscle specimen.

To address the question whether protein degradation occurs in the same fashion and in a similar time sequence within an individual muscle, as well as in different muscles and muscle types, we designed a pilot study analyzing muscle samples from three forensic autopsy cases with varying PMI and (morphological) degree of decomposition. Different locations of *M. vastus lateralis* were analyzed for intramuscular variance. To investigate intermuscular differences, degradation of *M. vastus lateralis* was compared with that of *M. temporalis* (jaw muscle) and *M. longitudinalis superior linguae* (tongue muscle), both expected to be less affected by interindividual conditions including training, injury, aging, etc. To analyze similarities and/or differences in muscle types, skeletal muscle (*M. vastus lateralis*) was compared with cardiac muscle (myocard) and smooth muscle (pyloric sphincter) samples.

## Material and methods

### Included cases

Muscle samples from three autopsy cases with varying degree of decomposition were analyzed in the course of this study (Table 1). Case A was a 33-year-old male, who got stabbed in a conflict. Despite resuscitation he died immediately after being transferred to hospital. Time between death and autopsy was about 57 h. The corpse presented fully developed rigor mortis. Due to substantial blood loss, livores were rare. No signs of decomposition were present. This case was classified as “fresh.” Case B is a 54-year-old male died of drowning. The exact time of death is unknown. He went missing by the end of December and was found early February at a hydroelectric power plant (water temperature approximately 3 °C). His corpse depicted reddish green discoloration of the skin and showed changes associated with postmortem immersion, such as washerwoman’s skin and slippage of the epidermis. There was no rigor mortis in the joints and postmortem lividity was pale. This case was classified as “early decomposition.” Case C is a 69-year-old male, who died of subdural hemorrhage. He was found in the bathroom of his apartment (room temperature approximately 20 °C) after a neighbor reported a foul smell to the police. He was last seen 10 days ago, which corresponded to the content of his postbox. His corpse showed a dark brown to greenish discoloration of the skin, colonization by blow fly larvae (including pupae), dry skin, epidermal loss especially at the trunk, and a partial loss of fingernails. Postmortem lividity could not be detected because of the coloration of the skin. There was no rigor mortis. This case was classified as “advanced decomposition.”

**Table 1:** Overview of the investigated cases

	A	B	C
Age	33	54	69
Sex	M	M	M
Height [cm]	172	184	181
Weight [kg]	52.8	95	79
Cause of death	Internal and external bleeding	Drowning	Subdural hemorrhage
PMI*/environment	2.4 days/4 °C cooling unit	Max. 42 days/3 °C water	Max. 10 days/20 °C apartment
Degree of decomposition	Fresh	Early decomposition	Advanced decomposition

*PMI postmortem interval

Notably, information on the PMI is highly imprecise for cases B and C, which is the case in most of the advanced decomposed corpses. Also, even though the maximum possible PMI for case B exceeds the one of case C, the higher environmental temperature distinctly accelerated the appearance of postmortem changes [16] in the latter. As the focus of the present study was to investigate eventual intraindividual differences, also cases with a lack of precise according information were included. However, no assignment of protein degradation events to specific PMIs or timeframes should be made, also given the small sample size.

### Sampling and sample preparation

In course of the autopsies, small muscle samples were collected from seven different body regions (Table 2), trimmed to approximately 5 × 5 × 5 mm, and snap frozen and stored in liquid nitrogen until further processing.

**Table 2:** Overview of the collected muscle samples

Muscle	Location/description	Abbreviation/s
*M. vastus lateralis*	Central in the muscle belly, from medium depth (central)	VL, VL cent, reference sample
*M. vastus lateralis*	Central in the muscle belly, close (1 cm) to the femur	VL med
*M. vastus lateralis*	Distal, close (1 cm) to the tendon	VL dist
*M. temporalis*	Central, from medium depth	T
*M. longitudinalis superior linguae*	Central, approx. 2 cm behind the tongue tip	LSL
Myocard	Left ventricle, close (2 cm) to the tip	MY (cardiac muscle)
Pyloric sphincter	Epithelium removed, 5 mm section of the ring muscle collected	PS (smooth muscle)

All samples were homogenized by cryogenic grinding and subsequent sonication. 10 × vol/wt RIPA buffer together with a protease inhibitor cocktail was used as lysis and extraction buffer. After centrifugation (10 min at 1000×*g*), the supernatant was transferred into a fresh test tube and frozen at − 20 °C until further use. Protein concentrations were determined using BCA assay. All samples were diluted to equal overall protein content prior to analysis.

### Analysis of protein degradation

Electrophoreses (SDS-PAGE) were run on 10% polyacrylamide resolving gels and 5% stacking gels, according to a standard protocol [6]. Thirty micrograms of total protein were prepared, denatured at 90 °C for 5 min, and inserted into the gel wells. Following electrophoresis, the proteins were transferred onto blotting membranes (polyvinylidene fluoride (PVDF)) and stored at − 20 °C until further use. Prior to immunolabeling, the membranes were blocked in blocking buffer (tris-buffered saline (TBS) with 1% BSA). The following primary antisera were used: mouse monoclonal anti-α-actinin, mouse monoclonal anti-α-tubulin, and mouse monoclonal anti-vinculin. HRP-conjugated polyclonal goat anti-mouse was applied as a secondary antibody. All antibodies were diluted in blocking agent and applied for 1 h. After antibody application, the membranes were extensively washed and rinsed in TBS. Antibody staining was visualized by application of chemiluminescence substrate and documented using a digital gel analysis system (Fusion FX7, Peqlab Biotechnology). Protein band intensities were measured using ImageJ software (ImageJ 1.45s, Java 1.6.0_20). Alterations, such as the disappearance of a native band or appearance of additional bands, were considered degradation events. Signals < 1% the intensity of the native bands were considered background and thus no band. For the depiction in the included figures, lanes were cropped, pasted, and adjusted for brightness and contrast.

## Results

All samples were collected and processed as intended. Photometric determination of the total protein concentration revealed sufficient amounts (2.0–6.5 μg/μl) for all samples. There were no signs of irregular electrophoresis runs or Western blot experiments detected, and all protein bands could be analyzed according to standard protocols.

### Intramuscular comparison

**Fig. 1:**
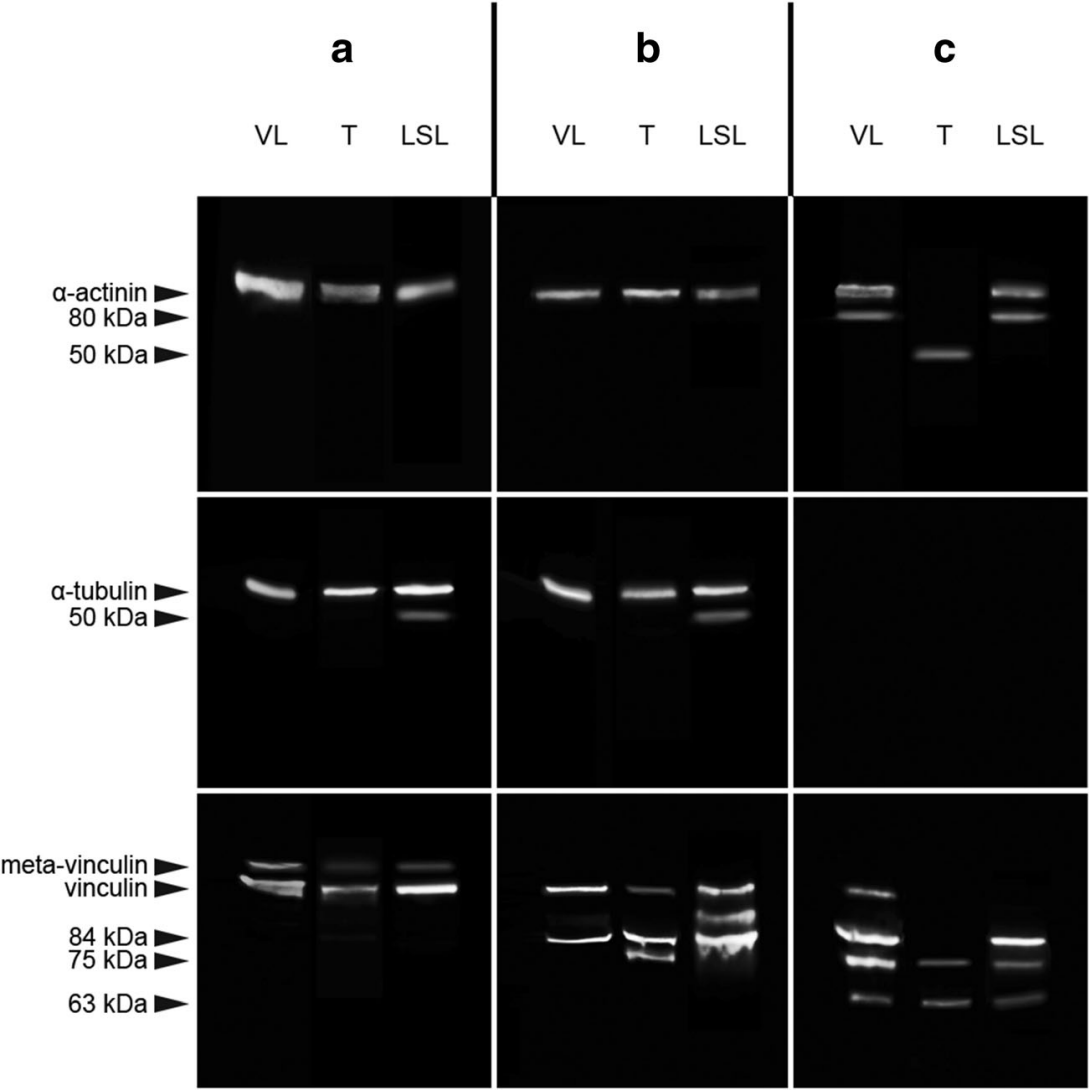
Degradation profiles of the investigated proteins in different locations, central (cent), medial (med), and distal (dist), within an individual muscle (*M. vastus lateralis* (VL)) of three autopsy cases (a–c). The cases were classified to different degrees of decomposition: a, “fresh”; b, “early decomposition”; and c, “advanced decomposition”

A native α-actinin band at approximately 100 kDa was present in all *M. vastus lateralis* samples tested in all three cases. Additionally, case C depicted an 80 kDa degradation product in all samples. Although the signal in the sample from the muscle center was comparably weak, it was clearly above the detection threshold and therefore considered present. In all samples collected from cases A and B, a distinct native α-tubulin band was detected at approximately 53 kDa. This band could not be found in any of the samples from case C. None of the samples depicted any α-tubulin degradation products. Native vinculin bands at approximately 117 kDa were detected in all samples analyzed. Although faint in all samples collected from case C, native vinculin bands were above the detection threshold. Meta-vinculin bands were exclusively found in all samples collected from case A. A vinculin degradation product at 84 kDa was present in all case B and C samples, but in none of the case A samples. Similarly, a 75 kDa degradation product was only detectable in samples from the two cases with early (case B, distal and medial *M. vastus lateralis* samples) and advanced decomposition (case C, all samples). A third vinculin degradation product of approximately 63 kDa was exclusively present in all samples collected from case C (Fig. 1).

### Intermuscular comparison of skeletal muscles

**Fig. 2:**
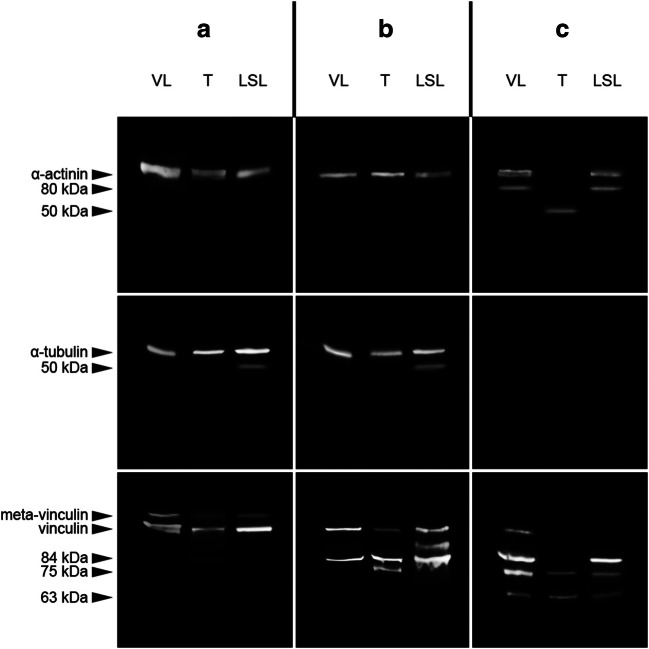
Degradation profiles of the investigated proteins in different skeletal muscles (*M. vastus lateralis* (VL), *M. longitudinalis superior linguae* (LSL), and *M. temporalis* (T)) of three autopsy cases with varying degree of decomposition (A, “fresh”; B, “early decomposition”; C, “advanced decomposition”)

A native α-actinin band (100 kDa) was present in all muscle samples of cases A and B. In case C, this band was only present in the samples collected from *M. vastus lateralis* and *M. longitudinalis superior linguae*, but not in *M. temporalis*. In the same samples, an α-actinin degradation product at 80 kDa was detected. In *M. temporalis* collected from case C, another degradation product at approximately 50 kDa was exclusively present. Native α-tubulin bands were present in all samples collected from cases A and B, but not in case C samples. A weak signal of an α-tubulin degradation product at 50 kDa was found in the *M. longitudinalis superior linguae* sample of case A, but none in other sample. The native vinculin band was detected in analyzed samples from cases A and B, but only in the *M. vastus lateralis* sample of case C. Meta-vinculin bands were exclusively found in all samples collected from case A. Degradation products at 84 kDa were detected in all samples of case B and the *M. vastus lateralis* and *M. longitudinalis superior linguae* sample of case C. Neither the *M. temporalis* sample of case C nor any of the samples from case A depicted this 84 kDa degradation product. A second vinculin degradation product at 75 kDa was detected in the *M. temporalis* sample from case B and all samples from case C. Ultimately, a 63 kDa degradation product was observed in all case C samples exclusively (Fig. 2).

### Intermuscular comparison of muscle types

**Fig. 3:**
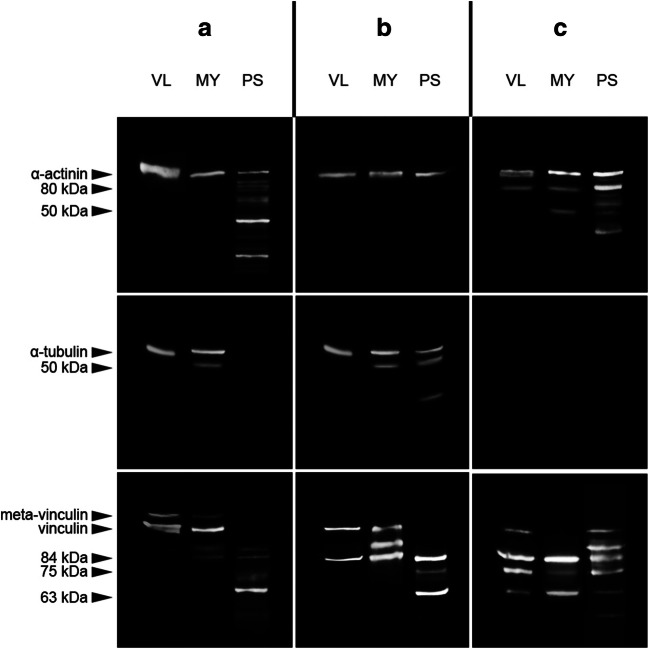
Degradation profiles of the investigated proteins in different muscle types, skeletal muscle (*M. vastus lateralis*), cardiac muscle (myocard), and smooth muscle (pyloric sphincter), of three autopsy cases with varying degree of decomposition (A, “fresh”; B, “early decomposition”; C, “advanced decomposition”)

A native α-actinin band at approximately 100 kDa was present in all samples tested. Degradation products at 80 kDa were detected in all samples collected from case C. Additionally, numerous protein bands of different molecular weights appeared in the pyloric sphincter samples from cases A and C. The myocard sample from case C depicted a single additional α-actinin degradation product at approximately 50 kDa. A native α-tubulin band was present in the skeletal muscle and cardiac muscle samples of cases A and B and the smooth muscle collected from case B. Additionally, a 50 kDa degradation product was detected in the myocard samples of cases A and B as well as the pyloric sphincter sample from case B. No α-tubulin bands were detected in any of the case C samples. A native vinculin band was present in the skeletal muscle samples of all three cases, as well as the myocard samples from A and B and the pyloric sphincter of case C. The skeletal muscle and cardiac muscle samples of case A were the only ones to depict meta-vinculin bands. A 84 kDa degradation product was present in all samples, except the skeletal muscle sample from case A. A fragment of 75 kDa was detectable in all samples from case C, as well as the pyloric sphincter sample from case B. Another degradation product with a molecular weight of 63 kDa was found in all samples taken from the pyloric sphincter and in all samples from case C. Additional, smaller fragments appeared in the pyloric sphincter sample of case A (Fig. 3).

## Discussion

Standardized protocols and awareness of the limitations of a method are of utmost importance for forensic application of PMI estimation methods. As such, it is inevitable to know whether there are intraindividual variations of (PMI) markers or measurement sites. Despite the small sample size of this study, the obtained results provide valuable data for methodic considerations and future research.

The progress of protein degradation in different sample sites within a specific muscle (*M. vastus lateralis*) revealed similar, well comparable, and yet not completely identical profiles. In one of the autopsy cases (B), a single protein fragment (75 kDa vinculin) was not detected in a sample collected from the central muscle region. A temperature effect due to differential postmortem body cooling can be excluded in this case, as the medial sample can be expected to remain at higher temperature for a longer time span, whereas the distal site cools faster [11]. As the postmortem development of degradation products has to be considered a gradual process, a signal can result above or beyond detection limit in different samples at a specific time point. This stochastic effect, however, represents a minor problem for PMI estimations as long as several proteins (and degradation products) are considered and appropriate mathematical models, including confidence intervals are applied. Notably, all other protein patterns from samples originating from different locations within *M. vastus lateralis* were identical within each case. Especially considering that extreme sampling positions were selected (1 cm distance to bone and tendon), the results suggest that intramuscular variations of protein degradation patterns can be largely disregarded. This endorses experimental designs with multiple samplings from the same muscle at different time points in research [e.g., 2, 16], as well as avoidance of injured or by any means affected locations (e.g., in dismembered body parts) for forensic PMI estimation [e.g., 17, 18].

In different skeletal muscles, discrete degradation patterns with increasing degree of morphological decomposition were detected. However, distinct differences between all investigated muscles were observed. Here, we compared muscles that can be considered less susceptible to in vivo variations such as training, injury, and atrophy (*M. temporalis* and *M. longitudinalis superior linguae*) and compared them with *M. vastus lateralis*, which served as a target muscle in previous studies [6, 8]. Especially *M. temporalis* showed advanced degradation compared with the other muscles in case C. The native bands of α-actinin and vinculin and some degradation products were lacking in this sample. Methodical errors can be excluded, as other degradation products were detected. Also the present 75 kDa vinculin band detected in the *M. temporalis muscle* of case B supports the findings of enhanced decomposition in this muscle, although, as mentioned above, this has to be viewed with some caution. *M. longitudinalis superior linguae* as well showed some deviations in degradation patterns compared with the other muscles. Specifically, this regards to an α-tubulin degradation product in the cases A and B, an approximately 100 kDa vinculin fragment in case B, and the loss of the native vinculin band in case C. Possible explanations for these differences include the varying composition on behalf of fiber types, connective tissue, and vascularization in skeletal muscles [14, 19, 20], according to their function (tonic, fast or slow twitch, etc.). Additionally, varying postmortem cooling rates in different parts of the body [11] can significantly affect protein degradation. Proteolysis is a metabolic process and as such highly dependent on (environmental) temperature [21]. While superficial structures and organs adjust to ambient temperature very quickly postmortem, internal sites in the body core can maintain elevated temperatures for more than 24 h [22]. A similar effect has also been described in context with electrical excitability of skeletal muscles [23]. However, the muscles selected as well as the small sample size of this study are not appropriate to test this hypothesis. Additional research, for example, comparing a distant, superficial muscle (e.g., *M. gastrocnemius*) to a deep proximal muscle (e.g., *M. psoas major*) in a larger sample size, would be necessary. Nevertheless, all tested skeletal muscles included in this study depicted decomposition-related changes that can potentially be used as markers for PMI estimation. While the (expected) fewer influence of training and aging in *M. temporalis* and *M. longitudinalis superior linguae* can be beneficial to reduce interindividual variations in estimation models, these muscles are definitely more difficult to investigate in non-autopsy settings (e.g., more complex sampling) compared with large limb muscles. However, varying decomposition speeds in different skeletal muscles have the potential to increase both the precision and applicable timeframe for PMI estimation when several muscles are analyzed.

Standard protocols, established for the analysis of skeletal muscle protein degradation [6], worked well also for cardiac and smooth muscle samples. Results suggest an acceptable transferability of protocols across investigated muscles, which should always be carefully validated prior to outcome generation as differing proteins and protein isoforms and antibody specificity might limit the application [24]. Myocard samples revealed successive protein degradation with advancing PMI of the corpses which all were generally similar to the patterns found in skeletal muscle. In comparison with vastus lateralis muscle, several signs of advanced decomposition were detected, such as α-actinin in case C, α-tubulin in case A and B, and vinculin in cases A and C. At no point, it was vice versa. This clearly suggests enhanced decomposition speed in cardiac muscle tissue compared with *M. vastus lateralis*. Whether this is due to the proximity to the body core and/or cell type specific metabolism can only be speculated. No valid assertions on postmortem protein degradation of smooth muscle can be made based on the obtained data. Protein analysis of the pyloric sphincter showed extreme deviations (a multitude of protein fragments of varying molecular weight in α-actinin and vinculin in cases A and C and a complete lack of bands in α-tubulin in case A) to all other muscles sampled. It can be assumed that this is a consequence of the proximity to aggressive acidic environment in the stomach and digestive enzymes, especially proteases from the pancreas, much rather than PMI-dependent decomposition. Interestingly, case B did not depict such dramatic changes. Here, we refrain from speculations to correlations with the time point of the last meal or the cause of death. Yet, analyses of the samples of case B indicate general applicability of the protocols to smooth muscle tissue. There is weak evidence for advanced decomposition of smooth muscle with the presence of α-tubulin and vinculin degradation products in case B compared with the other muscle types tested. Additional experiments using a more valuable source for investigations of protein degradation in smooth muscle without (major) influence by the gastrointestinal system (e.g., the *tunica media* of the aorta) and a larger sample size are necessary to test this.

In routine forensic application, easy sampling of target tissue is a crucial aspect. In the present comparative research study, all muscles and muscle regions were easily identified and accordingly sampled during autopsy regardless of decomposition stage of the corpses. However, for other study designs such as studies investigating multiple samples from an individual muscle in field research (e.g., at a human forensic taphonomy facility) as well as sampling in a non-autopsy setting (e.g., at a crime scene), most of the muscles used in this study suffer from restrictions. In fact, only *M. vastus lateralis* and (with some limitations) *M. temporalis* can be considered for according research, as the sampling procedure would most likely have a manageable influence on the rest of the body. During sample preparation, no aberrations were detected. Different rigidity due to varying content of cytoskeletal elements and connective tissue [20] which in turn could deteriorate the homogenization process and/or the obtainable protein content was not observed. Also on behalf of postmortem decomposition (especially in case C), protein concentration measurements revealed sufficient amounts in all samples indicating the applicability of the method also in advanced degradation stages.

## Conclusion

Postmortem degradation of muscle proteins is a highly conserved process within an individual muscle as well as (with varying rate) throughout different muscles and muscle types. Intramuscular variances are limited, supporting validity and replicability, whereas intermuscular differences offer a possibility to further improve the method. Large skeletal muscles of the limbs offer beneficial opportunities for research and are comparably easy to access (e.g., by muscle biopsy) also in scenarios where no autopsy is carried out. At the same time, other muscles have advantages on behalf of smaller interindividual variations. Analyzing specific muscles or a thoughtful combination of several muscles can ultimately improve both the precision and the temporal applicability for a reliable method to estimate the PMI.
